# Localization and orientation of heavy-atom cluster compounds in protein crystals using molecular replacement

**DOI:** 10.1107/S0907444912046008

**Published:** 2013-01-19

**Authors:** Sven O. Dahms, Miriam Kuester, Carsten Streb, Christian Roth, Norbert Sträter, Manuel E. Than

**Affiliations:** a Protein Crystallography Group, Leibniz Institute for Age Research – Fritz Lipmann Institute (FLI), Beutenbergstrasse 11, D-07745 Jena, Germany; b Institute of Inorganic Chemistry II, Department of Chemistry and Pharmacy, Friedrich-Alexander-University Erlangen-Nürnberg, Egerlandstrasse 1, D-91058 Erlangen, Germany; c Institute of Bioanalytical Chemistry, Center for Biotechnology and Biomedicine, Universität Leipzig, D-04103 Leipzig, Germany

**Keywords:** experimental phasing, heavy-metal cluster, hexasodium α-metatungstate, molecular replacement, death receptor 6

## Abstract

Heavy-atom clusters (HA clusters) containing a large number of specifically arranged electron-dense scatterers are especially useful for experimental phase determination of large complex structures, weakly diffracting crystals or structures with large unit cells. Often, the determination of the exact orientation of the HA cluster and hence of the individual heavy-atom positions proves to be the critical step in successful phasing and subsequent structure solution. Here, it is demonstrated that molecular replacement (MR) with either anomalous or isomorphous differences is a useful strategy for the correct placement of HA cluster compounds. The polyoxometallate cluster hexasodium α-metatungstate (HMT) was applied in phasing the structure of death receptor 6. Even though the HA cluster is bound in alternate partially occupied orientations and is located at a special position, its correct localization and orientation could be determined at resolutions as low as 4.9 Å. The broad applicability of this approach was demonstrated for five different derivative crystals that included the compounds tantalum tetradeca­bromide and trisodium phosphotungstate in addition to HMT. The correct placement of the HA cluster depends on the length of the intramolecular vectors chosen for MR, such that both a larger cluster size and the optimal choice of the wavelength used for anomalous data collection strongly affect the outcome.

## Abstract 2

A new approach is presented that allows the efficient localization and orientation of heavy-atom cluster compounds used in experimental phasing by a molecular replacement procedure. This permits the calculation of meaningful phases up to the highest resolution of the diffraction data.

## 1. Introduction   

Solving the phase problem is a bottleneck in protein crystallography. Unless a useful model for molecular replacement (MR) is available, experimental phasing is the method of choice (Adams *et al.*, 2009 ▶). The particular challenge in experimental phasing is to obtain sufficiently well diffracting derivative crystals that yield measurable isomorphous or anomalous differences. Once the substructure of the hetero­atoms has been solved, the resulting information can be used to calculate phases for the protein content of the whole crystal (reviewed, for example, in Taylor, 2010 ▶). Many approaches, often based on difference Patterson or anomalous difference Patterson methods or on direct methods, have been developed. Relevant popular and easy-to-use computer programs in this context are *SHELX* (Sheldrick, 2008 ▶), *Shake-and-Bake* (Miller *et al.*, 2007 ▶), *RSPS* (Knight, 2000 ▶), *SHARP* (Bricogne *et al.*, 2003 ▶), *HySS* (Grosse-Kunstleve & Adams, 2003 ▶), *SOLVE* (Terwilliger & Berendzen, 1999 ▶), *Phaser* (McCoy *et al.*, 2007 ▶), *CRUNCH* (de Graaff *et al.*, 2001 ▶) and *BP*3 (Pannu *et al.*, 2003 ▶; Pannu & Read, 2004 ▶); alternatively, the translation function can be used to determine single heavy-atom positions (Vagin & Teplyakov, 1998 ▶). To generate an isomorphous or an anomalous signal that is detectable against the background, a certain number of heavy atoms (or anomalous scatterers) per amino-acid residue need to be introduced into (or natively present in) the protein crystal (Boggon & Shapiro, 2000 ▶). Consequently, for large proteins, protein complexes and asymmetric units containing multiple molecules, very large numbers of such heteroatoms are required. The resulting heavy-atom substructures are highly complex, complicating the localization of the heavy-atom-binding sites. This barrier can often be overcome by using heavy-metal clusters (HA clusters) as the derivatization agent (Thygesen *et al.*, 1996 ▶; Mueller *et al.*, 2007 ▶). An ensemble of several heavy atoms is introduced into a protein crystal for each bound HA cluster molecule. At low resolutions (6–8 Å) the individual atoms of the HA clusters scatter in phase and behave as ‘super heavy-atoms’. However, the total scattering contribution of the spherically arranged HA cluster atoms is greater compared with an equal number of randomly distributed heavy atoms (Dauter, 2005 ▶). Very strong anomalous or isomorphous signals are generated in such derivative crystals and the centres of mass of HA clusters are detected with high sensitivity, for example in an anomalous difference Patterson map.

Heteropolytungstate clusters containing 12–30 spherically arranged octahedral WO_6_ subunits have been used to solve the structures of, for example, fumarase C (Weaver *et al.*, 1995 ▶), RNA polymerase (Fu *et al.*, 1999 ▶), the LDL receptor (Rudenko *et al.*, 2003 ▶), the proteasome core complex (Löwe *et al.*, 1995 ▶) and ribosomal subunits (see, for example, Ban *et al.*, 1999 ▶; Clemons *et al.*, 2001 ▶). The most frequently used HA cluster is probably the tantalum tetradecabromide cluster Ta_6_Br^2+^_12_ (TaB; Knäblein *et al.*, 1997 ▶; Dauter, 2005 ▶). This compound contains an octahedral heavy-metal core of six Ta atoms and has been applied to solve the structures of, for example, furin (Henrich *et al.*, 2003 ▶), RuBisCO and trans­cetolase (Schneider & Lindqvist, 1994 ▶) and the proteasome core complex (Löwe *et al.*, 1995 ▶), as well as RNA polymerase (Zhang *et al.*, 1999 ▶; Cramer *et al.*, 2000 ▶).

If the preferred orientation is unknown and the HA clusters are treated as super heavy-atoms, phase information is restricted to a resolution equal to their diameter (Dauter, 2005 ▶). To improve the phase quality, the specifically structured heavy-atom ensemble can be placed in a random orientation at the centre of mass (Schneider & Lindqvist, 1994 ▶). Alternatively, the spherically arranged heavy-atom shell of HA-cluster compounds can be approximated by spherical averaging to improve the phase quality at low and medium resolution (see, for example, Schluenzen *et al.*, 2000 ▶; Oubridge *et al.*, 2009 ▶; Fu *et al.*, 1999 ▶). This approach also accounts for possible disordered binding modes of HA clusters (Schluenzen *et al.*, 2000 ▶). However, for efficient model building the phase information obtained from HA cluster derivatives needs to be extended to higher resolution. In several studies, noncrystallographic symmetry operators have been used in combination with solvent flattening to extend the resolution limit of the experimental phase information (see, for example, Brandstetter *et al.*, 2001 ▶; Henrich *et al.*, 2003 ▶; Szczepanowski *et al.*, 2005 ▶; Kroemer & Schulz, 2002 ▶). Low-resolution phases obtained from HA cluster derivatives have also been used to solve more complex heavy-atom substructures of single-heavy-atom derivatives. In most cases, additional single-heavy-atom derivatives were finally used to calculate experimental phases to high resolution (see, for example, Ban *et al.*, 1999 ▶; Cramer *et al.*, 2000 ▶; Wahl *et al.*, 2000 ▶; Singleton *et al.*, 2004 ▶).

If HA cluster derivative crystals are directly used to calculate experimental phases, correct placement of the individual cluster atoms is essentially required to overcome this resolution barrier. However, the correct orientation of HA clusters is a challenging task, especially when only weakly diffracting derivative crystals are available. The identification of the individual Ta atoms of TaB-derivative crystals using automated structure-solution programs required highly resolved diffraction data (Banumathi *et al.*, 2003 ▶; Pasternak *et al.*, 2008 ▶). Below 2.6 Å resolution determination of the individual Ta sites failed, even when a truncated data set with a high-resolution limit of 1.8 Å was used (Pasternak *et al.*, 2008 ▶). Alternatively, Knablein and coworkers applied a two-step procedure for orientation of TaB (Knäblein *et al.*, 1997 ▶). In the first step, the HA cluster was localized in anomalous Patterson maps at low resolution. Once the centre of mass had been identified, rigid-body refinement was performed in *X-­PLOR* to orient the HA cluster, using the crystallographic *R* factor and the Patterson correlation value as the target function. This procedure succeeded with isomorphous differences at 3 Å resolution, using truncated data sets resolved to 2.2 or 2.3 Å resolution, and was also adapted for the correct placement of naturally occurring [Fe_4_S_4_] clusters using dispersive differences (Dias *et al.*, 1999 ▶). Another approach, which however also requires sufficient resolution of the diffraction data, is that implemented in* SHARP* (Bricogne *et al.*, 2003 ▶), which uses initial spherical averaging to approximate the cluster shape during phasing and subsequent identification of the individual HA sites from log-likelihood-gradient maps (residual/LLG maps).

Here, we use an MR-based approach for the localization and orientation of HA cluster compounds even at low resolution (down to 4.9 Å). This strategy was applied to determine the HA substructure of the polyoxometallate cluster compound hexasodium α-metatungstate (HMT) in derivatized crystals of the extracellular cysteine-rich region of death receptor 6 (DR6). The structure of DR6 was originally determined using aurate-derivative crystals in a multiple-wavelength anomalous dispersion (MAD) experiment (Kuester *et al.*, 2011 ▶) or by sulfur single-wavelength anomalous dispersion (Ru *et al.*, 2012 ▶). The crystals of DR6 derivatized with HMT proved to be a challenging model system for investigating experimental phase determination with HA-cluster compounds. This MR-based strategy of substructure determination succeeded with different HA clusters, a number of protein crystals and even in complicated cases, and proves to be especially useful for diffraction data at intermediate resolution when other methods fail.

## 2. Materials and methods   

### 2.1. Hexasodium α-metatungstate derivative crystals of DR6   

DR6 was cloned, prepared and crystallized as described by Kuester *et al.* (2011 ▶). Crystals were grown within 2 d from 0.1 *M* sodium phosphate pH 6.1, 30%(*w*/*v*) PEG 4000, 0.3 *M* ammonium acetate and were derivatized by soaking for 1 h in 0.1 *M* sodium citrate pH 6.0, 0.3 *M* ammonium acetate, 30%(*w*/*v*) PEG 4000 containing 5 m*M* hexasodium α-metatungstate (HMT; Jena Bioscience). Native and derivative crystals were flash-cooled in liquid nitrogen for data collection.

### 2.2. HA cluster derivative crystals of hexagonal lysozyme   

For the preparation of hexagonal hen egg-white lysozyme (HEWL) crystals, the previously described batch procedure (Haas, 1967 ▶) was adapted for sitting-drop vapour diffusion as follows: HEWL (Roth) was dissolved in water, mixed with reservoir solution in a 3:2 ratio (drop volume 5 µl) and equilibrated in 24-well Cryschem plates (Hampton Research) at 293 K against 1 ml reservoir solution. The reservoir solution consisted of a 1:10 mixture of 0.5 *M* tricine pH 7.4 and saturated sodium nitrate in 10%(*v*/*v*) aqueous acetone. Crystals grew within 24–48 h to maximal dimensions of 1.0 mm. Trisodium phosphotungstate (TPT; Jena Bioscience) was added to the reservoir solution to a final concentration of 2.5 m*M*, resulting in phase separation. The upper phase was immediately removed and derivative crystals were prepared by 2 h soaking in the upper phase. Prolonged storage of the soaking solution as well as prolonged soaking times resulted in lower occupancies of the HA cluster in the crystals, indicating ongoing degradation reactions. Tantalum tetradeca­bromide (TaB; Jena Bioscience) was nearly insoluble in the reservoir solution. Hence, a suspension of 1%(*w*/*v*) TaB in reservoir solution was acidified with 1 *M* sodium acetate pH 3.8 until the colour of the soluble phase turned dark green. The supernatant of this suspension was added stepwise to a 1 µl drop of reservoir solution containing several crystals until the crystals adopted a dark green colour. The crystals were treated with Paratone-N (Hampton Research) for cryoprotection prior to flash-cooling in liquid nitrogen.

### 2.3. HA cluster derivative crystals of styrol monooxygenase (StyA1)   

StyA1 from *Rhodococcus opacus* 1CP was cloned, expressed and purified as described by Tischler *et al.* (2010 ▶). An ammonium sulfate precipitation of purified StyA1 was dissolved in buffer consisting of 20 m*M* Tris pH 7.3, 150 m*M* NaCl, 5 m*M* DTT and applied onto a Superdex 200 column (GE Healthcare) equilibrated with the same buffer. The StyA1-containing fractions were pooled and concentrated to 8 mg ml^−1^. Crystals were grown in 0.1 *M* sodium cacodylate pH 6.0–7.0, 0.2 *M* potassium chloride, 14–16%(*w*/*v*) PEG 8000 within 4 d at 292 K. Suitable crystals were transferred into a stabilization solution consisting of 0.1 *M* sodium cacodylate, 0.2 *M* potassium chloride with a 2%(*w*/*v*) elevated PEG concentration supplemented with 10%(*v*/*v*) 1,2-ethanediol as a cryoprotectant as well as 10 m*M* TaB. Crystals were equilibrated for 24 h in this buffer prior to flash-cooling in liquid nitrogen.

### 2.4. Measurement and processing of diffraction data   

Diffraction data were collected from native and derivative DR6 crystals using an Enraf–Nonius FR591 rotating-anode X-­ray generator (Bruker AXS) equipped with FOX mirror optics (Xenocs) and a MAR 345 image-plate detector (MAR Research), resulting in the SIRAS data. MAD and SAD data sets were collected from the same DR6 crystal and from lysozyme crystals using synchrotron radiation (MAD data on BL14.2 and SAD data on BL14.1 at BESSY II, Helmholtz-Zentrum Berlin; Mueller *et al.*, 2012 ▶). Prior to measurement of the MAD and SAD data sets, an X-ray fluorescence emission spectrum was collected to determine the exact energetic locations of the white lines of tantalum and tungsten. MAD data were collected in the order peak, inflection point, high-energy remote. The data were integrated with *MOSFLM* (v.7.0.1; Leslie & Powell, 2007 ▶), scaled with *SCALA* (v.3.3.16; Evans, 2006 ▶) and further processed using programs from the *CCP*4 suite (v.6.1.13; Winn *et al.*, 2011 ▶). Diffraction data for StyA1 were collected on BL14.1 at BESSY at the Ta peak wavelength, which was determined prior to data collection from the X-ray fluorescence emission spectrum. The data were integrated with *XDS* (v.06/2010; Kabsch, 2010*a* ▶) and scaled with *XSCALE* (v.06/2010; Kabsch, 2010*b* ▶). The statistics of the processed data sets are shown in Table 1 ▶. The native and derivative data sets of the DR6 crystals were scaled together in *SCALEIT* and isomorphous differences for the MR searches were calculated with *SFTOOLS*.

The diffraction data shown in Table 1 ▶ are available upon request from the authors and have been deposited at http://www.fli-leibniz.de/www_pxg/supp-mat/.

### 2.5. Localization and orientation of HA clusters and phase calculation   

MR searches for the orientation and localization of the HA clusters in the DR6 and lysozyme crystals were performed in *MOLREP* (v.10.2.35; Vagin & Teplyakov, 2010 ▶) using *CCP*4 v.6.1.13 and the *CCP*4*i* interface v.2.0.6. Isomorphous or anomalous differences were used instead of structure-factor amplitudes. If the HA clusters were located on a symmetry axis (HMT derivative of DR6 and TaB derivative of lysozyme), the packing function had to be disabled. In the case of the fragment of mouse ubiquitin-activating enzyme (MUAE), an overall *B* factor of 85 Å^2^ was applied for the MR search, guided by the reported Wilson *B* factor of the diffraction data (Szczepanowski *et al.*, 2005 ▶). The atomic coordinates of α-metatungstate (ABOCIE; Niu *et al.*, 2004 ▶), phosphotungstate (BIGLIN; Huang *et al.*, 2004 ▶) and the dodecabromohexa­tantalum cation (AFASUV; Vojnović *et al.*, 2002 ▶) were obtained from the Cambridge Structural Database (CSD; Allen, 2002 ▶) and used as search models. To test the performance of this approach, additional MR searches were conducted using isomorphous or anomalous differences to the resolution limits given in Table 2 ▶. These resolution limits were applied using the RESMAX keyword in *MOLREP*. For MR searches at the Ta peak wavelength only the Ta coordinates were used as a search model, whereas a combined model consisting of both the Ta and the Br coordinates was used as a search model at the Br peak wavelength.

Subsequently to MR, experimental phases were calculated in *SHARP* v.2.6.0 (Bricogne *et al.*, 2003 ▶). Phases were calculated using the native and the MAD peak data sets in the case of DR6–HMT, the W peak and the Ta peak data sets in the case of lysozyme and the Ta peak data set in the case of StyA1 (Table 1 ▶). For HMT and TaB, the atomic positions obtained from MR were used directly and only *B*-factor and occupancy refinement was performed in *SHARP*. For TPT, the structure of the initial search model in MR differed from the final degradation product found in the crystals and positional refinement was used for all sites. Anomalous difference maps indicated the presence of additional single-heavy-atom sites. These sites were located by stepwise residual/LLG-map analysis, refined and included in the phase calculation in *SHARP*. For comparison, phase calculation was also performed based on spherical averaging of the HA clusters in *SHARP* (Bricogne *et al.*, 2003 ▶) using the SPHCLUSTER keyword as recommended in the *SHARP* manual (http://www.globalphasing.com/sharp/manual/appendix3.html). The cluster definitions of TaB and TPT as provided in *SHARP* were used; for HMT, the cluster properties were adapted to the slightly smaller radius of 7.0 Å. For the spherically averaged HA clusters the positions (originally the centre of mass), the occupancies and the *B* factors were refined. Residual/LLG-map analysis was performed in *SHARP* either by inspection of peak lists or by visual inspection of the map.

The heavy-atom search in *SHELXC*/*D* (Sheldrick, 2010 ▶) was performed using the *CCP*4*i* interface with varying numbers of expected sites and up to 100 000 trials. For the TaB cluster interatomic distances of 1.5 and 2.0 Å were tested and for the larger tungstate clusters interatomic distances of 2.5 and 3.0 Å were tested.

Density modification was performed in *SOLOMON* (Abrahams & Leslie, 1996 ▶) using the *SHARP* density-modification control panel in *SUSHI* v.3.8.0. *DM* (Cowtan, 1994 ▶) was used for NCS averaging. The respective NCS operators were initially determined from the self-rotation function calculated in *MOLREP* and were subsequently refined in *GETAX* (Vonrhein & Schulz, 1999 ▶).

### 2.6. Evaluation of the phase quality   

To achieve a relative comparison of the phase quality, different sets of experimental phases were compared with model phases calculated from the atomic coordinates. Because of significant non-isomorphism between the high-resolution data set (PDB entry 3qo4; Kuester *et al.*, 2011 ▶) and the HMT-derivative data sets, the high-resolution structure of DR6 had to be subjected to additional rounds of refinement. The DR6 structure was refined to the native in-house data set by rigid-body refinement in *REFMAC* (v.5.5.0109; Murshudov *et al.*, 2011 ▶), giving *R* and *R*_free_ factors of 22.7% and 25.5%, respectively. In addition, the DR6 structure, including the W atoms, was refined to the high-energy remote derivative data set by rigid-body refinement in *REFMAC*, model building in *MAIN* (Turk, 1992 ▶) and positional refinement in *CNS* (v.1.3; Brunger, 2007 ▶), giving *R* and *R*_free_ factors of 24.5% and 27.6%, respectively. Initial rounds of rigid-body and positional refinement for the HA cluster derivative structures of lysozyme and StyA1 were performed in *CNS*, giving *R* and *R*_free_ factors of 23.5% and 28.2%, respectively, for TaB–lysozyme, 28.0% and 30.6%, respectively, for TPT–lysozyme and 21.7% and 25.7%, respectively, for StyA1. The individual occupancies and *B* factors of the HA clusters were refined in *CNS*. *SFTOOLS* was used to calculate the cosine of the phase difference between the model phases (native DR6 and DR6–­HMT) and the respective experimental protein phases (SIRAS and MAD). The values are plotted in Fig. 2 as a function of the resolution (using intervals comprising similar numbers of reflections). Map correlation coefficients were calculated with *OVERLAPMAP*, comparing maps calculated from experimental and model phases, and are shown in Fig. 3. Molecular graphics were prepared with *PyMOL* (http:// www.pymol.org).

## 3. Results   

### 3.1. Preparation of hexasodium α-metatungstate derivative crystals of DR6   

**Figure 1:**
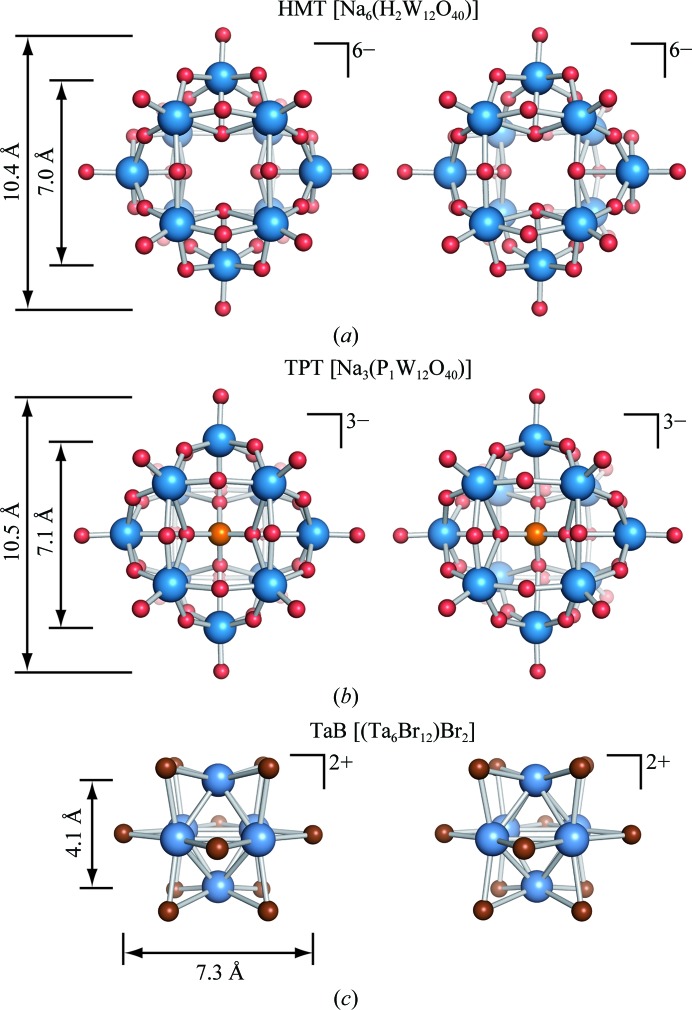
Structural comparison of the HA cluster compounds used in this study. The HA clusters are shown as stereo ball-and-stick representations. (*a*) Hexasodium α-metatungstate (HMT). (*b*) Trisodium phosphotungstate (TPT). In these tungstate clusters, large blue spheres represent tungsten, medium-sized orange spheres represent phosphorus and small red spheres represent oxygen. (*c*) Hexatantalum tetradecabromide (TaB): large blue spheres represent tantalum, medium-sized brown spheres represent bromine.

We successfully obtained isomorphous DR6 derivative crystals by soaking them in stabilizing solution supplemented with 5 m*M* HMT. This HA cluster is composed of 12 W atoms and has a similar structure to the more commonly used TPT (Fig. 1 ▶). The phosphate core of the latter is replaced by four water molecules in HMT, resulting in a greater overall charge of −6 (Figs. 1 ▶ *a* and 1 ▶ *b*). Both clusters are characterized by an internal tetrahedral symmetry, but differ in the W—O—W bond angles as a result of a differently isomerized WO_4_^−^ substructure. HMT is soluble in acidic, neutral and slightly basic aqueous solutions with high or low ionic strength. In contrast, the other HA cluster compounds tested (TaB and TPT) showed lower solubility under the tested conditions. As low solubility is often a limiting factor for the practical use of derivatization agents, this observation favours the broad application of HMT (Hastings & Howarth, 1992 ▶).

Tungsten displays a strong anomalous diffraction of 5.6 electrons at the Cu *K*α wavelength and a strong white line at its *L*_III_ absorption edge (1.2147 Å). Hence, successful derivatization of DR6 crystals with HMT could be monitored by a significantly increased anomalous correlation coefficient (CC_anom_) of the in-house data. The unit-cell parameters of the native and derivative crystals (SIRAS in Table 1 ▶) showed only slight differences and the corresponding data sets indicated a sufficient degree of isomorphism (see Supplementary Material^1^). Three additional data sets were collected from the same derivative crystal at the W *L*_III_ absorption edge to perform a three-wavelength MAD experiment (MAD in Table 1 ▶).

### 3.2. Orientation and localization of HMT by molecular replacement   

To orient the HA cluster and thus to localize the individual W-atom positions of HMT, we applied molecular replacement (MR) in *MOLREP* (Vagin & Teplyakov, 2010 ▶). The coordinates of the Cambridge Structural Database (CSD) entry ABOCIE (Allen, 2002 ▶) were directly used as the search model. *MOLREP* was run with default settings *via* the *CCP*4*i* interface, disabling the packing and the scoring system. Instead of structure-factor amplitudes, either the isomorphous differences of the in-house data sets (SIRAS in Table 1 ▶) or the anomalous differences of the MAD peak data set (‘peak’ in Table 1 ▶) were used. To test the performance of this procedure at different resolutions, the respective data sets were cut into 0.5 Å steps and the MR calculations were repeated. Correct solutions were well separated from noise by significantly higher score and contrast (TFcnt) values in *MOLREP* (Table 2 ▶) and were found for isomorphous as well as anomalous differences down to resolutions as low as 4.4 and 4.9 Å, respectively. In addition, we could also identify ‘partial solutions’ represented by intermediate scoring values. In this case the correct centre of mass but an incorrect orientation of the HA cluster was identified, resulting in incorrect placement of the individual W atoms. Such ‘partial solutions’ were obtained down to 5.4 Å resolution using anomalous differences.

### 3.3. Evaluation of experimental phases obtained by the HA cluster MR procedure   

**Figure 2:**
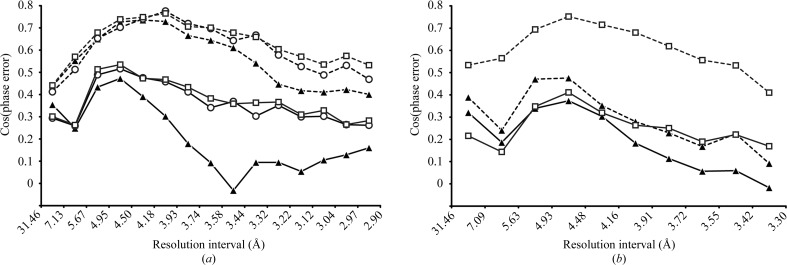
Comparison of the phase quality obtained for the HMT-derivative DR6 crystals. The cosine of the phase error between the experimental protein phases and the final model phases is shown as function of the resolution (see §[Sec sec2]2 for details). Filled triangles represent phases calculated by spherical averaging in *SHARP* (SPHCLUSTER option), whereas open symbols represent phases calculated from the individual tungsten positions of the MR searches calculated at 2.9 Å resolution (squares) and 4.4 Å resolution (circles). The resolution limits refer to the data used for the heavy-atom cluster orientation by MR; the final phases were always calculated up to the resolution maximum of the data sets (Table 1 ▶). Phases calculated from the respective heavy-atom models are connected by solid lines; phases after density modification are connected by broken lines. (*a*) Comparison of phases determined in a three-wavelength MAD experiment. (*b*) Comparison of phases determined by a SIRAS experiment.

**Figure 3:**
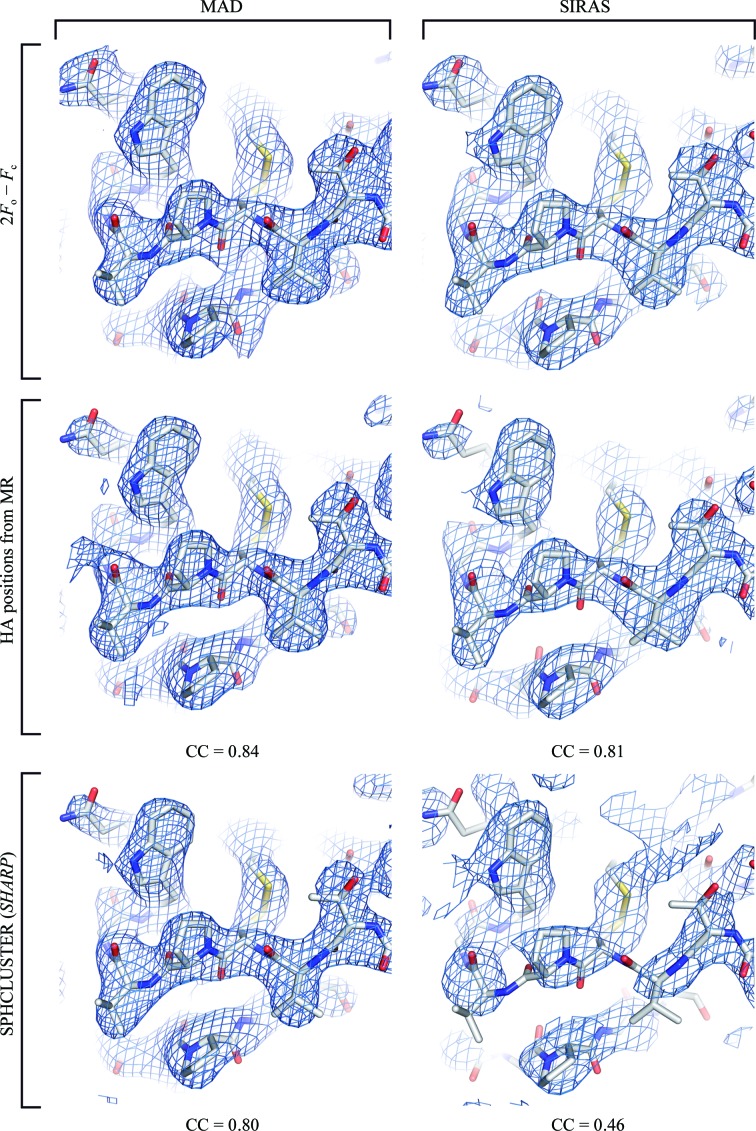
Comparison of experimental electron-density maps calculated for HMT-derivative DR6 crystals. The electron-density maps (contoured at 1σ) were calculated from experimental phases (MAD experiment at 2.9 Å resolution or SIRAS experiment at 3.3 Å resolution) after solvent flattening and are shown in comparison with the 2*F*_o_ − *F*_c_ difference Fourier map calculated from the final protein model (stick representation). The experimental phases were either derived from the individual tungsten positions of the MR solution at 2.9 Å resolution (MAD) or at 3.4 Å resolution (SIRAS) or by spherical averaging in *SHARP* (SPHCLUSTER option) at 2.9 Å resolution (MAD) or at 3.4 Å resolution (SIRAS). The map correlation coefficient (CC) is given in comparison to the respective 2*F*_o_ − *F*_c_ map for each experimental electron-density map.

The localization and orientation of the HMT cluster by MR yields the individual W-atom positions, allowing the calculation of phases to high resolution. For comparison, experimental phases were calculated by spherical averaging at the centre of mass (the SPHCLUSTER option in *SHARP*). The experimental phases determined in *SHARP* were evaluated in relation to reference phases calculated from the DR6 structure (Fig. 2 ▶). To calculate reference phases of good quality, DR6 structures were initially refined to the tungsten high-energy remote data set and to the native in-house data set, showing *R*/*R*_free_ values of 24.5/27.6% and 22.7/25.5%, respectively. The best phase quality was clearly observed when the individual tungsten sites of the MR solutions were used for phase calculation. In contrast, the initial phases obtained by spherical averaging were of low quality, especially beyond 4.5 Å resolution. For the MAD experiment, a general improvement of the phase quality was observed after solvent flattening. However, in the resolution range beyond 3.5 Å the phases calculated based on spherical averaging still showed significantly more error in comparison to phases calculated based on the MR solution. Correspondingly, the experimental electron-density map in the latter case shows more detail (for example, around Thr148, Val149 and Trp154) and is comparable to the 2*F*_o_ − *F*_c_ difference Fourier map (Fig. 3 ▶).

The in-house diffraction data are of lower quality than the MAD data, as indicated by the lower resolution limit of 3.3 Å of the native data set and other parameters. As expected, the experimental phases obtained in the SIRAS experiment resulted in phases of lower quality than those obtained from the MAD experiment. However, the threshold of information required to achieve an interpretable electron-density map upon density modification was not reached in the SIRAS experiment when spherical averaging was used. In contrast, the phases calculated based on the MR solutions are drastically improved upon solvent flattening and resulted in an easily interpretable electron-density map. Consequently, if weaker diffraction data and hence less information are available for phase calculation, obtaining the optimal heavy-atom substructure and hence initial phases of high quality represent the bottleneck for successful structure solution. The observed differences in the phase quality are in good agreement with the map correlation coefficients calculated from experimental and model phases (Fig. 3 ▶).

The W-atom sites were used for phase calculation without further positional refinement. Consequently, the phase error is expected to increase greatly with an increase in the positional error of the MR solutions. Interestingly, a comparison of phases calculated with MR solutions obtained at high and low resolution (2.9 and 4.4 Å, respectively) revealed comparable good quality. Apparently, the positional accuracy of the MR procedure, once the solution has been found, is only slightly decreased at 4.4 Å resolution and the heavy-atom sites derived from the MR solutions at low resolution are thus equally suited for phase calculation. This observation is in agreement with the distribution of the scoring values in *MOLREP* with resolution, reaching maxima in the region of 4 Å.

### 3.4. Multiple occupied states of HMT in DR6 crystals   

**Figure 4:**
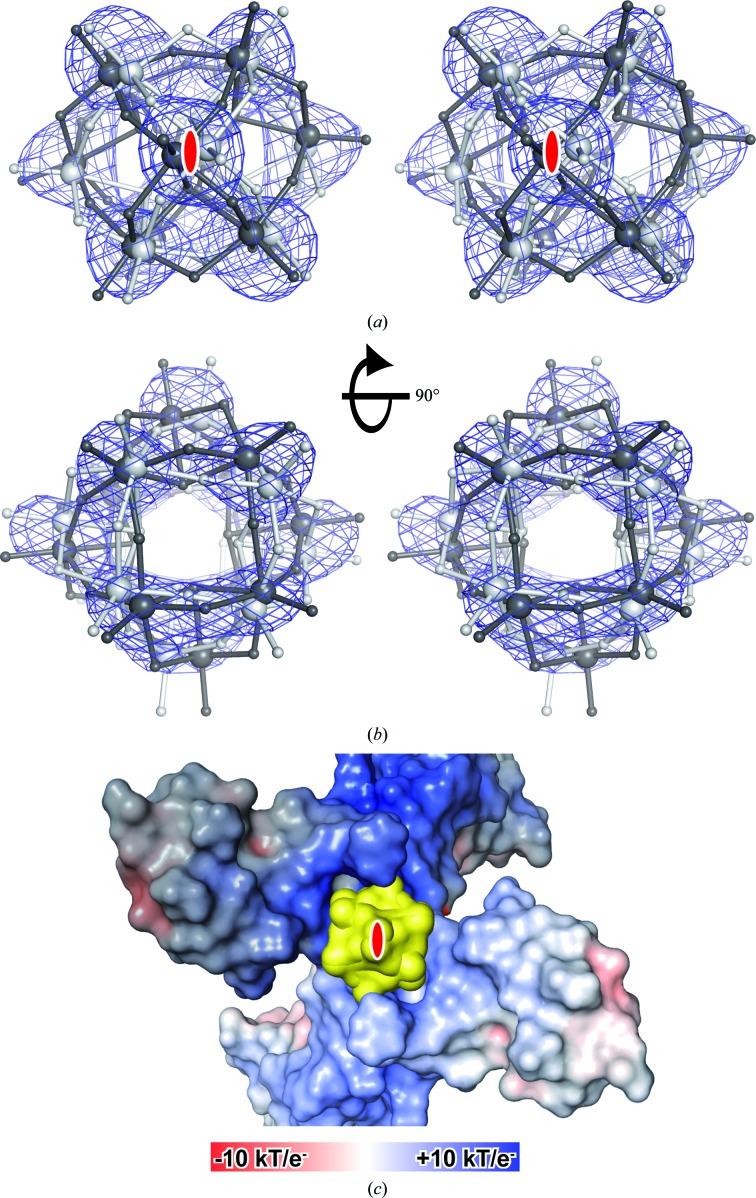
HMT is bound at a special position in multiple occupied states in DR6 derivative crystals. (*a*, *b*) Stereo representations of the anomalous difference Fourier map (calculated with phases from the final DR6 model and anomalous differences from the W peak data set; contoured at 5σ) together with a ball-and-stick representation of the HMT cluster (large spheres represent the W atoms). The two symmetry-related HA cluster molecules are shown in dark grey and light grey. (*a*) A view along the crystallographic twofold symmetry axis (marked in red) and (*b*) a view perpendicular to it. (*c*) Two symmetry-related DR6 molecules are shown as a surface representation (coloured according to their calculated electrostatic potential) together with the bound HMT cluster molecules (yellow). Light and dark colours distinguish between the symmetry-related DR6 protomers. The location of the twofold symmetry axis is indicated in red.

A closer inspection of the obtained MR solutions revealed the localization of HMT on a twofold crystallographic symmetry axis (Fig. 4 ▶). However, the local twofold symmetry axis of the HA cluster does not coincide with the twofold crystallographic symmetry axis and hence breaks the crystallographic symmetry. Correspondingly, the individual W-atom positions of the two symmetry-related HA cluster molecules showed an increasing divergence along the twofold crystallographic symmetry axis. This divergence between the symmetry-related HA cluster molecules is coincident with a blurring of the anomalous difference density map (Fig. 4 ▶ *b*). This condition can be interpreted as two alternative partially occupied orientations of HMT inside the crystal. Each of the two observed HA cluster orientations forms stronger contacts to one of the DR6 molecules and hence belongs to a different asymmetric unit. Binding occurs at a positively charged pocket formed by two symmetry-related protein molecules. This tendency to bind at special positions, also observed in other studies (*e.g.* Ladenstein *et al.*, 1987 ▶), complicates the treatment of the HA cluster during experimental phase determination. The multiple occupied state of HMT is accompanied by a low average occupancy of ∼33% (calculated by occupancy refinement in *CN*S), reducing the anomalous or isomorphous signal compared with a fully occupied HA-cluster site. As a result of this binding state, the individual tungsten positions were unstable during positional heavy-atom refinement in *SHARP*. However, the solutions obtained by MR fitted very well to the two different HA-cluster binding states. Consequently, these coordinates could directly be entered for phasing in *SHARP* without further positional refinement.

### 3.5. Application of MR to substructure determination in TPT- and TaB-derivative crystals of hen egg-white lysozyme   

Encouraged by the successful application of MR to substructure determination in HMT-derivative DR6 crystals, we tested the applicability of this approach to a variety of HA cluster derivatives. We first prepared and analyzed HA cluster derivatives of the hexagonal crystal form of hen egg-white lysozyme (HEWL). This crystal form shows large solvent channels, which allow the unhindered diffusion of HA cluster compounds inside the crystal (PDB entry 2fbb; Brinkmann *et al.*, 2006 ▶). TPT and TaB were not very soluble in the crystallization condition, requiring an optimized soaking procedure (see §[Sec sec2]2 for details). In contrast, the highly soluble HMT resulted in rapid degradation of the lysozyme crystals, probably as a consequence of binding of HMT to lysozyme and the resulting destruction of crystal contacts.

**Figure 5:**
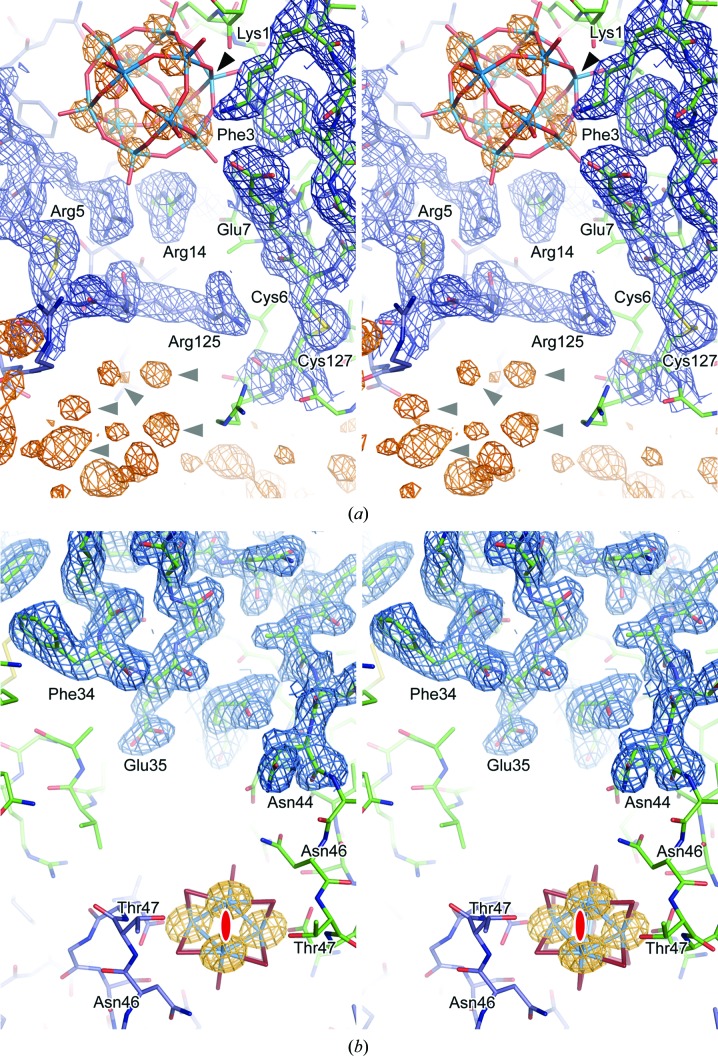
Derivative crystals of hexagonal HEWL with TPT and TaB. A stereo representation of a section of the solvent-flattened experimental electron-density map is shown calculated from SAD phases (blue mesh, contoured at 1σ) together with the anomalous difference Fourier map of tungsten and tantalum (orange mesh contoured at 5σ) for (*a*) HEWL–TPT (1.9 Å resolution) and (*b*) HEWL–TaB (1.9 Å resolution), respectively. HEWL is shown in stick representation; blue and purple C atoms mark two symmetry-related molecules. (*a*) TPT oriented by MR is shown in stick representation with light blue W and red O atoms. Owing to degradation, only 11 W atoms reside within the heavy-atom cluster, whereas additional peaks were found in the anomalous Fourier map. Black and grey arrowheads mark the positions of the missing and additional W atoms, respectively. (*b*) The TaB cluster at binding site 1 was found by MR at 1.8 Å resolution and is shown in stick representation. The twofold crystallographic symmetry axis is marked in red.

The two bound clusters, TPT and TaB, were successfully oriented and localized by MR, as clearly indicated by the scoring values in *MOLREP* (Table 2 ▶). A closer inspection of the TPT-derivative crystals revealed low occupancy of the HA cluster. Occupancy refinement in *CNS* revealed an average occupancy of 10%; similarly, the average anomalous occupancy was refined in *SHARP* to only 13%. Even more surprisingly, only 11 tungsten positions were confirmed in the anomalous difference Fourier map (Fig. 5 ▶ *a*). This observation indicates the formation of the so-called lacunary anion [PW_11_O_39_]^7−^, a degradation product of this cluster compound (Müller *et al.*, 1998 ▶). Additionally, 13 weakly occupied single heavy atoms were identified in the asymmetric unit, again hinting at degradation of TPT (Fig. 5 ▶ *a*). These sites were clustered at distances varying from around 2 to 4 Å, but did not show any obvious order. The observed degradation of TPT during derivatization of the crystals is expected to interfere with the MR search. Indeed, the search procedure worked well down to a resolution of 2.2 Å, but failed abruptly at resolutions of 2.3 Å or lower. Nonetheless, experimental phases could finally be calculated to high resolution in a SAD experiment. Initially employing the original MR solution, we first identified the correct W_11_ substructure and subsequently located the additional low-occupancy tungsten sites employing residual/LLG-map analysis in *SHARP* (Fig. 5 ▶ *a*).

For the TaB cluster derivative, one strongly occupied binding site as well as a second more weakly occupied binding site were identified by MR, corresponding to anomalous occupancies after heavy-atom refinement in *SHARP* of 45% and 25%, respectively (the occupancies refined in *CNS* were 33% and 22%, respectively). TaB is bound at a twofold symmetry axis at each site, perfectly matching the crystallo­graphic symmetry (note that the location of the cluster necessarily causes a formal reduction in the occupancy). Phase calculation in *SHARP* employing a SAD phasing scheme resulted in an experimental electron-density map of good quality when both HA cluster sites were used (Fig. 5 ▶ *b*). However, even phasing with only the highly occupied TaB site enabled the calculation of an interpretable electron-density map (data not shown).

The orientation and localization of TaB were also determined at lower resolution using the Ta peak data set. However, the positional errors of the solutions increased at resolutions below 3.3 Å. Correspondingly, the peaks of the Ta anomalous density map and the Ta atoms of the MR solution showed a high r.m.s.d. of 0.83 Å at 3.8 Å resolution compared with 0.23 Å at 1.8 Å resolution. This difficulty was overcome by using a data set measured at the bromine absorption edge and including the Br atoms in the search model (Table 2 ▶). Although the anomalous contribution of the Br atoms is much lower compared with tantalum at the bromine peak wavelength, the TFcnt value in *MOLREP* drastically increased at 3.8 Å resolution from 2.2 to 4.8 and the r.m.s.d. to the peaks of the Ta anomalous density map decreased to 0.24 Å. This observation is explained by the larger interatomic distances of the Br atoms (∼7 Å) compared with the Ta atoms (∼4 Å) of TaB. These interatomic distances define the low-resolution limit at which the individual atoms begin to scatter in phase and hence appear as a super heavy-atom. Consequently, larger interatomic distances apparently enable the determination of the orientation of HA cluster compounds down to lower resolutions and can be achieved by the choice of the cluster compound and the wavelength used for the diffraction experiment.

### 3.6. Application of MR to substructure determination in TaB-derivative StyA1 crystals   

**Figure 6:**
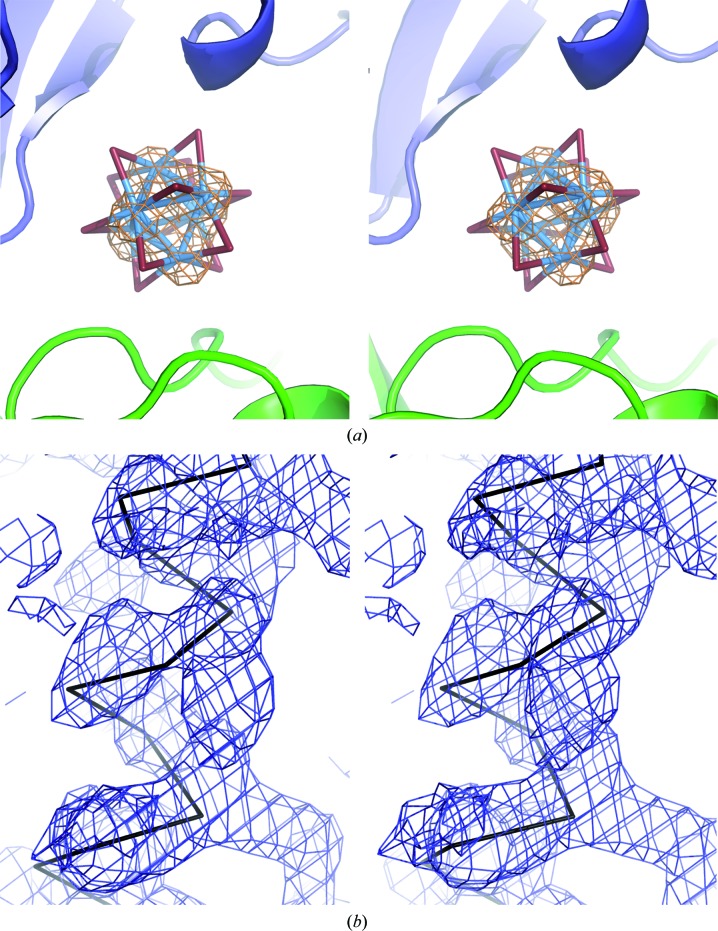
TaB-derivative crystals of StyA1. Stereo representations of (*a*) the anomalous difference Fourier map of tantalum (orange mesh, contoured at 5σ; 2.4 Å resolution) at the TaB cluster binding site 1 (in stick representation) together with two adjacent protein molecules in ribbon representation and (*b*) the experimental electron-density map after solvent flattening and twofold NCS averaging calculated from SAD phases (blue mesh, contoured at 1σ; 2.4 Å resolution) together with the C^α^ trace of the final model in black are shown.

For experimental phase determination of StyA1, TaB-derivative crystals were obtained together with other single-heavy-atom derivatives (to be published; Table 1 ▶). However, location of TaB failed with *SHELXD* although the data were resolved to 2.4 Å resolution. Employing the MR approach, a TaB site was readily identified (Fig. 6 ▶ *a*). Correct and incorrect solutions are clearly discriminated by highest score values of 0.188 and 0.087, respectively (Table 2 ▶). Using these Ta sites in a SAD experiment, a second TaB site was located by residual/LLG-map analysis in *SHARP*. Occupancy refinement in *CNS* revealed that site 2 showed a much lower occupancy (∼30%) compared with site 1 (∼60%). Finally, all Ta sites together were used in a SAD experiment to obtain an interpretable electron-density map after solvent flattening and twofold NCS averaging (Fig. 6 ▶ *b*).

### 3.7. Application of MR to substructure determination in derivative crystals of a fragment of the mouse ubiquitin-activating enzyme   

**Figure 7:**
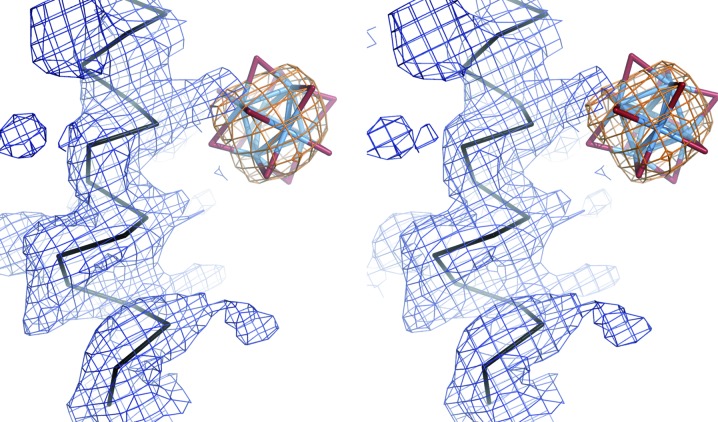
TaB-derivative crystals of MUAE. A stereo representation of a section of the experimental electron-density map is shown after phase extension to 2.8 Å resolution, solvent flattening and threefold NCS averaging calculated from SAD phases (blue mesh, contoured at 1σ) together with the anomalous difference Fourier map of tungsten (orange mesh, contoured at 5σ). The C^α^ trace of the final model (PDB entry 1z7l) is shown as ribbon representation (black). The TaB cluster at binding site 1 was found by MR at 3.3 Å resolution and is shown in stick representation.

Another challenging test case for experimental phase determination was the X-ray structure of a fragment of the mouse ubiquitin-activating enzyme (MUAE; PDB entry 1z7l; Szczepanowski *et al.*, 2005 ▶). The final structure was resolved to 2.8 Å resolution. The derivative crystals contained three TaB clusters with very high average *B* factors (∼87 Å^2^) and very low occupancies (15, 18 and 19%). Consequently, these TaB sites appeared as single peaks in the anomalous difference map, but their orientation could still be determined by the shape of the anomalous difference map. Initial attempts to place TaB by MR with the Ta peak data at 3.3 Å resolution revealed only one of the two stronger sites, probably because of the weak anomalous signal beyond 5 Å resolution (Szczepanowski *et al.*, 2005 ▶). To improve the quality of the input data, we combined the anomalous data of all three MAD data sets by summation. In addition, an overall *B* factor of 85 Å^2^ was applied to the search model and the packing function was enabled. Running a sequential MR search in *MOLREP* with these settings, the correct three solutions were identified as indicated by an increasing score value, which otherwise stagnated at the score value of site 1. Interestingly, two of these solutions fitted well to the shape of the anomalous difference map (Fig. 7 ▶). One cluster was placed in a random orientation. Nonetheless, good phases were calculated in a SAD experiment and an interpretable electron-density map was obtained after phase extension to 2.8 Å resolution (high-energy remote data set), solvent flattening and NCS averaging (Fig. 7 ▶).

### 3.8. Comparison of the MR approach to other methods for substructure determination of HA clusters   

To compare the performance of the MR approach with standard methods, we repeated the HA substructure determinations for all of the examples listed in Table 2 ▶ with *SHELXD* and with spherical averaging in *SHARP*. If initial phases of sufficient quality were obtained by spherical averaging, the individual heavy-atom sites were determined by residual/LLG-map analysis in *SHARP*.

At high resolution, the individual heavy-atom sites of the lysozyme-derivative crystals were readily identified in *SHELX* (Table 3 ▶). However, for less well resolved data (≤2.4 Å) the correct HA substructures could not easily be determined in this way.

As shown for the HMT-derivative DR6 crystals, spherical averaging results in poor phase information at high resolution. However, given diffraction data of sufficient resolution, the single-heavy-atom sites could be determined for lysozyme–TaB and for StyA1–TaB in consecutive residual/LLG-map completion steps. At lower resolutions (*e.g.* for lysozyme–TaB) and/or lower occupancies (*e.g.* site 2 of StyA1–TaB) simple inspection of peak lists was insufficient to determine the orientation of HA clusters as the signal was lower than the noise. In some cases the HA clusters could still be placed as rigid bodies by visual inspection of LLG maps, but for DR6–HMT, lysozyme–TPT and MUAE–TaB the single-heavy-atom sites and hence the orientation of the HA clusters could only be determined by MR.

## 4. Discussion   

### 4.1. Hexasodium α-metatungstate as a heavy-atom compound for protein crystallography   

We have applied the hexasodium α-metatungstate cluster (Na_6_[H_2_W_12_O_40_]; HMT) for experimental phasing of the structure of DR6 by SIRAS and MAD. The HA cluster is composed of 12 W atoms forming a tetrahedral arrangement with a diameter of 7 Å, geometric properties that are quite similar to those of the trisodium phosphotungstate (TPT) cluster. HMT showed superior solubility in the DR6 reservoir solution compared with the other tungstate clusters tested. Similar behaviour was observed in the crystallization condition of hexagonal lysozyme crystals, although exposure to HMT resulted in decomposition of these crystals. However, its solubility and stability over a wide pH range and at higher ionic strengths is a great advantage of the use of HMT as a heavy-atom compound in protein crystallography (Hastings & Howarth, 1992 ▶; Garman & Murray, 2003 ▶).

HMT was shown to bind in a positively charged pocket between two symmetry-related DR6 molecules. This observation is in agreement with the binding preference reported for other tungstate clusters (Bashan & Yonath, 2008 ▶), all of which carry a negative net charge. In the DR6 crystals, HMT is located on a crystallographic twofold axis. The tendency of HA clusters to bind at crystallographic symmetry axes has also been described in other studies (see, for example, Ladenstein *et al.*, 1987 ▶; Rudenko *et al.*, 2003 ▶). The coincidence of the internal symmetry of the HA cluster with crystallographic symmetry elements can help to orient the HA cluster correctly once the centre of mass has been identified. However, in the DR6 crystals HMT breaks the twofold crystallographic symmetry. It adopts multiple orientations bound to one of two symmetry-related DR6 molecules. This condition complicated the orientation of the HA cluster, because the effective anomalous signal is reduced and the resulting electron-density map is blurred. Despite these drawbacks, the HA cluster was successfully localized and oriented by MR, even at resolutions lower than 4 Å. The resulting electron-density map was of exceptionally high quality and comparable to the final 2*F*_o_ − *F*_c_ electron-density map calculated from the final protein model.

### 4.2. Application of MR to determination of the orientation and localization of HA clusters in protein crystals   

If HA clusters are treated as super heavy point scatterers, meaningful experimental phases are limited to resolutions lower than their diameter (Dauter, 2005 ▶). Given that HA clusters are bound in an ordered fashion, phasing to high resolution requires their correct orientation and hence the placement of the individual heavy atoms. Encouraged by the successful application of MR to the orientation and localization of HMT in DR6 derivative crystals, we compared the performance of this approach with standard methods. Five test systems were evaluated, comprising four different proteins as well as three different HA clusters. At high resolution, the heavy-atom substructures of the HEWL TPT- and TaB-derivative crystals were readily identified using *SHELXD*, consistent with previous reports (Banumathi *et al.*, 2003 ▶). Similarly, *SOLVE* was reported to identify the heavy-atom substructure of TaB down to 2.6 Å resolution (Pasternak *et al.*, 2008 ▶). Compared with the treatment of HA clusters as point scatterers with large *B* factors (super heavy-atoms), the approximation of their heavy-atom substructures by spherical averaging can improve the quality of experimental phases (see, for example, Schluenzen *et al.*, 2000 ▶; Oubridge *et al.*, 2009 ▶; Fu *et al.*, 1999 ▶). Initial phases obtained by spherical averaging can then be used as a starting point to identify the orientation of HA clusters by residual/LLG-map analysis as implemented in *SHARP* (Bricogne *et al.*, 2003 ▶). At low resolution, only manual interpretation of the residual/LLG maps allowed the correct placement and orientation of the HA cluster.

However, for the DR6–HMT and HEWL–TPT crystals spherical averaging finds only limited application. In both cases we found heavy-atom substructures that largely deviated from the spherically averaged model. Consequently, the orientation of both HA clusters could not be determined by residual/LLG maps based on initial phases. Using the individual heavy-atom positions from MR solutions, we observed initial phases for DR6–HMT of tremendously improved quality. Determination of the individual tungsten sites was a prerequisite to obtain interpretable electron density in a SIRAS experiment with HMT-derivative DR6 crystals at 3.3 Å resolution. In the TPT-derivative crystals, a low-occupancy W_11_ degradation product of the HA cluster was successfully identified using the complete TPT structure as a search model. The resulting phases to high resolution were used to identify an additional unusual arrangement of 13 W atoms by residual/LLG-map analysis in *SHARP* (Bricogne *et al.*, 2003 ▶).

MR-based orientation is expected to be especially useful if HA clusters with larger heavy-atom cores are used, *e.g.* tungsten clusters of ≥12 atoms. Owing to the larger inter­atomic distances of the W atoms, the orientation of HMT by MR is still possible at resolutions lower than 4.0 Å, whereas the orientation of the smaller TaB is hardly deducible at this resolution. The performance of the MR-based HA cluster orientation reached a maximum between 3.4 and 4.4 Å resolution for HMT, which is readily explained by the unique scattering properties of the spherically arranged heavy atoms (Dauter, 2005 ▶). A maximum of the scattering contribution is reached at a specific resolution depending on the diameter of the HA cluster. Correspondingly, tungstate clusters with 12 or more heavy atoms are especially suited for experimental phase determination of weakly diffracting crystals, a condition that is often correlated with large asymmetric units and high solvent contents of the crystals. Such crystals also tend to contain larger solvent channels, a prerequisite for permeation by HA cluster compounds.

Another consideration is the use of anomalous diffraction data in MR searches. Unge and coworkers correlated, for example, the anomalous rotation functions of sulfur substructures with the conventional rotation function to improve model placement (Unge *et al.*, 2011 ▶). However, the inherent flexibility of proteins and the resulting positional error of the search models decreased the performance of the sulfur anomalous rotation functions in this study (Anderson *et al.*, 1996 ▶; Unge *et al.*, 2011 ▶). Such complications were not observed for the various HA cluster compounds used in this study, all of which were composed of very rigid groups of strong anomalous scatterers. Furthermore, the choice of the wavelength for collection of the anomalous diffraction data largely influenced the limiting resolution for HA cluster placement. Only data collected at the bromine absorption edge permitted the precise placement of TaB at a resolution of 3.8 Å, which is in excellent agreement with the larger inter­atomic vectors between the Br atoms compared with the Ta atoms.

Consequently, MR employing either isomorphous or anomalous differences as structure factors is a very useful tool for the orientation and localization of HA clusters in protein-derivative crystals. This approach works over a broad resolution range and even in complicated test cases in which standard methods fail. Based on the MR solutions, phases were calculated up to the resolution limit of the diffraction data without the need for additional single-heavy-atom derivatives for all of the investigated examples. The classical MR search in *MOLREP* directly combines two steps: the initial orientation of the search model and its subsequent location by calculation of the rotation and translation functions (Vagin & Teplyakov, 1997 ▶, 2010 ▶). This strategy directly yields the individual heavy-atom positions as it combines the ‘classical’ heavy-atom search step to determine its centre of mass as well as the orientation of the HA cluster. A reverse order of the two steps as, for example, applied to position protein models in electron-density maps by an initial spherical averaged phased translation function (SAPTF) and a subsequent phased rotation function (Vagin & Isupov, 2001 ▶) or as applied by Knäblein *et al.* (1997 ▶) seems preferential at first thought. The simplicity and efficiency of use clearly support the classical MR search procedure applied here.

A typical MR search of this kind is also easily calculated within seconds by non-experts in *MOLREP* and showed a similar if not a better performance in comparison to the more laborious procedures such as that described by Knäblein *et al.* (1997 ▶). Hence, it might be useful to implement an MR-based search option for HA clusters in future versions of program suites for experimental phase determination.

## Supplementary Material

Supporting information file. DOI: 10.1107/S0907444912046008/wd5185sup1.pdf

## Figures and Tables

**Table 1:** Data-collection statistics Values in parentheses are for the highest resolution shell.

	DR6					Lysozyme			StyA1
	Native	HMT				TPT	TaB		TaB
	SIRAS	SIRAS	MAD			W peak	Ta peak	Br peak	Ta peak
			Peak	Inflection point	Remote				
Data-collection temperature (K)	100								
Wavelength ()	1.54	1.54	1.21461	1.21506	1.21340	1.21471	1.25496	0.91977	1.2250
Resolution limit ()	3.3 (3.483.30)	3.0 (3.163.00)	2.9 (3.062.90)	2.9 (3.062.90)	2.9 (3.062.90)	1.9 (2.001.90)	1.8 (1.901.80)	1.8 (1.901.80)	2.4 (2.472.41)
Space group	*P*6_1_22					*P*6_1_22			*P*2_1_
Unit-cell parameters									
*a* ()	77.88	77.34	77.27	77.24	77.27	86.51	86.35	86.39	61.67
*b* ()									78.62
*c* ()	183.70	184.64	184.71	184.64	184.68	68.22	68.86	68.87	75.36
()									107.52
Completeness (%)	99.9 (100.0)	99.7 (99.6)	99.8 (99.8)	99.3 (99.8)	99.8 (99.8)	100.0 (100.0)	98.5 (97.1)	99.5 (99.0)	99.2 (96.3)
Anomalous completeness (%)		100.0 (100.0)	99.9 (100.0)	99.4 (100.0)	99.6 (100.0)	100.0 (100.0)	98.9 (97.6)	99.7 (99.3)	98.1 (89.4)
*R*_merge_ (%)	18.2 (40.1)	10.4 (34.2)	7.8 (30.7)	6.5 (24.2)	8.6 (35.3)	8.0 (39.4)	6.0 (31.2)	6.6 (38.2)	8.8 (53.3)
*R*_p.i.m._ (%)	6.9 (14.3)	8.2 (27.1)	5.6 (22.6)	4.7 (17.7)	6.2 (25.9)	3.3 (16.2)	2.7 (14.2)	3.0 (17.3)	7.3 (33.6)
*I*/(*I*)	10.9 (5.2)	12.2 (3.9)	14.6 (4.5)	16.9 (5.7)	13.3 (4.0)	20.2 (6.1)	25.9 (6.6)	23.4 (6.0)	9.67 (1.67)
Multiplicity	7.7 (8.7)	4.5 (4.7)	5.0 (5.2)	5.0 (5.2)	5.0 (5.2)	12.8 (12.9)	10.6 (10.8)	10.7 (10.9)	2.4 (2.0)
CC_anom_ †		0.144	0.858	0.679	0.339	0.715	0.886	0.490	0.732

† Anomalous difference correlation between half-sets computed in *SCALA* (Evans, 2006 ▶; not available for the native DR6 data set).

**(a):** DR6HMT.

			Highest *MOLREP* TFcnt/score		
	Resolution ()	Solution	Correct	Centre of mass	Incorrect
Anomalous differences†	2.9	Yes	8.8/0.271	4.0/0.177	2.9/0.146
	3.4	Yes	9.9/0.377		3.5/0.195
	3.9	Yes	11.8/0.445	7.7/0.396	3.1/0.240
	4.4	Yes	7.8/0.468	5.8/0.396	
	4.9	Yes	3.7/0.437	3.8/0.433	
	5.4	(Yes)		4.6/0.528	2.3/0.466
	5.9				2.0/0.372
Isomorphous differences‡	3.4	Yes	6.9/0.269	3.9/0.212	2.5/0.169
	4.4	Yes	4.7/0.397	3.6/0.343	
	5.4				2.0/0.470

**(b):** HEWLTPT.

			Highest *MOLREP* TFcnt/score		
	Resolution ()	Solution	Correct	Centre of mass	Incorrect
W peak§	1.9	Yes	5.8/0.331		2.8/0.199
	2.2	Yes	4.8/0.292		2.2/0.154
	2.3				3.4/0.147

**(c):** HEWLTaB.

			Highest* MOLREP* TFcnt/score		
	Resolution ()	Solution	Site 1	Site 2	Centre of mass
Ta peak	1.8	Yes	7.7/0.023	2.7/0.009	
	2.8	Yes	6.7/0.341	3.0/0.154	1.9/0.121
	3.3	Yes	9.8/0.358		3.4/0.237
	3.8	(Yes)			2.2/0.404
Br peak	3.8	Yes	4.8/0.283		1.8/0.222

**(d):** StyA1TaB.

		Highest *MOLREP *TFcnt/score		
Resolution ()	Solution	Site 1	Site 2	Incorrect
2.6	Yes	3.82/0.188		3.20/0.087

**(e):** MUAETaB§

			Highest *MOLREP* TFcnt/score			
	Resolution ()	Solution	Site 1	Site 2	Site 3	Incorrect
Step 1	3.3	Yes	2.12/0.169			1.66/0.134
Step 2				1.63/0.200		
Step 3					1.82/0.227	

† MR search performed with the anomalous differences of the tungsten peak data set.

‡ MR search performed with isomorphous differences calculated from the native and derivative data sets obtained at the home source ( = 1.54).

§ Sequential search.

**Table 3:** Comparison of heavy-atom search methods

			Search method				
			MR search procedure			SPHCLUSTER	*SHELXD*
	Resolution ()	Total No. of HA clusters/single HA sites	Clusters located (MR run)	Sites found (MR run)	Sites added after LLG-map completion	Sites found after LLG-map completion	Solution found for single HA sites
DR6HMT, MAD	2.9	1/12	1	12	0	0	
DR6HMT, SIRAS	3.4	1/12	1	12	0	0	
LysozymeTPT	1.9	1/11	1	11	13	0	Yes
	2.2		1	11	13	0	Yes
LysozymeTaB	1.8	2/12	2	12	0	12	Yes
	2.8		2	12	0	12	
	3.8		1	6	6†	12†	
StyA1TaB	2.4	2/12	1	6	6†	12†	
MUAETaB	3.3	3/18	3	12	0	0	

† Visual inspection of LLG maps was required.

## Footnotes

1 Supplementary material has been deposited in the IUCr electronic archive (Reference: WD5185). Services for accessing this material are described at the back of the journal.
