# Supplementary material for: Localization and orientation of heavy-atom cluster compounds in protein crystals using molecular replacement
Source: Acta Crystallogr D Biol Crystallogr. 2013 Jan 19;69(Pt 2):284–97. doi: 10.1107/S0907444912046008 (PMC3565441; doi:10.1107/S0907444912046008)
Supplement: Supplementary file 1 [file d-69-00284-sup1.pdf]

**Supplementary Material**

R-factors calculated between native and derivative DR6 data sets:

| | R-factor between data sets (calculated in SCALEIT): | | | |
|---|---|---|---|---|
| Resolution (Å) | Native/ HMT-SIRAS | Native/ MAD-peak | Native/ MAD-inflection point | Native/ MAD-remote |
| 18.0 | 0.692 | 0.632 | 0.514 | 0.659 |
| 10.4 | 0.735 | 0.658 | 0.460 | 0.693 |
| 8.0 | 0.411 | 0.352 | 0.314 | 0.369 |
| 6.8 | 0.292 | 0.243 | 0.212 | 0.260 |
| 6.0 | 0.278 | 0.251 | 0.224 | 0.271 |
| 5.4 | 0.272 | 0.239 | 0.225 | 0.259 |
| 5.0 | 0.271 | 0.234 | 0.215 | 0.249 |
| 4.6 | 0.266 | 0.232 | 0.218 | 0.249 |
| 4.4 | 0.237 | 0.212 | 0.209 | 0.228 |
| 4.1 | 0.226 | 0.213 | 0.207 | 0.221 |
| 3.9 | 0.230 | 0.213 | 0.213 | 0.226 |
| 3.7 | 0.224 | 0.219 | 0.217 | 0.228 |
| 3.6 | 0.229 | 0.224 | 0.224 | 0.233 |
| 3.5 | 0.253 | 0.242 | 0.238 | 0.250 |
| 3.3 | 0.274 | 0.251 | 0.242 | 0.247 |
| Overall: | 0.285 | 0.259 | 0.237 | 0.272 |

| | R-factor between data sets(calculated in SCALEIT): | | |
|---|---|---|---|
| Resolution (Å) | HMT-SIRAS/ MAD-peak | HMT-SIRAS/ MAD-inflection point | HMT-SIRAS/ HMT-remote |
| 18.0 | 0.050 | 0.125 | 0.039 |
| 10.4 | 0.067 | 0.137 | 0.040 |
| 8.0 | 0.088 | 0.152 | 0.078 |
| 6.8 | 0.103 | 0.185 | 0.094 |
| 6.0 | 0.093 | 0.141 | 0.089 |
| 5.4 | 0.097 | 0.141 | 0.098 |
| 5.0 | 0.083 | 0.121 | 0.082 |
| 4.6 | 0.082 | 0.111 | 0.072 |
| 4.4 | 0.073 | 0.096 | 0.073 |
| 4.1 | 0.072 | 0.092 | 0.074 |
| 3.9 | 0.066 | 0.083 | 0.069 |
| 3.7 | 0.081 | 0.094 | 0.086 |
| 3.6 | 0.089 | 0.109 | 0.092 |
| 3.5 | 0.099 | 0.117 | 0.099 |
| 3.3 | 0.124 | 0.149 | 0.123 |
| 3.2 | 0.144 | 0.180 | 0.140 |
| 3.1 | 0.153 | 0.190 | 0.146 |
| 3.0 | 0.158 | 0.201 | 0.150 |
| Overall: | 0.099 | 0.132 | 0.096 |

| | R-factor between data sets(calculated in SCALEIT): | | |
|---|---|---|---|
| Resolution (Å) | MAD-peak/ MAD-inflection point | MAD-peak/ MAD-remote | MAD-inflection point/ MAD-remote |
| 18.0 | 0.081 | 0.042 | 0.134 |
| 10.4 | 0.08 | 0.042 | 0.108 |
| 8.0 | 0.058 | 0.04 | 0.08 |
| 6.8 | 0.062 | 0.051 | 0.082 |
| 6.0 | 0.056 | 0.06 | 0.085 |
| 5.4 | 0.059 | 0.054 | 0.079 |
| 5.0 | 0.053 | 0.045 | 0.068 |
| 4.6 | 0.043 | 0.046 | 0.066 |
| 4.4 | 0.039 | 0.042 | 0.055 |
| 4.1 | 0.04 | 0.045 | 0.055 |
| 3.9 | 0.037 | 0.043 | 0.053 |
| 3.7 | 0.046 | 0.055 | 0.056 |
| 3.6 | 0.052 | 0.06 | 0.069 |
| 3.5 | 0.063 | 0.074 | 0.081 |
| 3.3 | 0.079 | 0.082 | 0.098 |
| 3.2 | 0.093 | 0.11 | 0.121 |
| 3.1 | 0.106 | 0.116 | 0.136 |
| 3.0 | 0.117 | 0.13 | 0.161 |
| 2.9 | 0.139 | 0.138 | 0.175 |
| Overall: | 0.065 | 0.066 | 0.087 |
